# Quantitative evaluation of an optimized Time of Flight Magnetic Resonance Imaging procedure using a phantom setup to simulate aqueous humor flow

**DOI:** 10.1016/j.dib.2018.03.018

**Published:** 2018-03-09

**Authors:** Thomas Wecker, Christian van Oterendorp, Wilfried Reichardt

**Affiliations:** a Eye Center, Medical Center – University of Freiburg, Faculty of Medicine, University of Freiburg, Germany; b Department of Ophthalmology, Georg-August University Hospital, Göttingen, Germany; c Medical Physics, Department of Radiology, Faculty of Medicine, University of Freiburg, Germany; d German Consortium for Translational Cancer Research (DKTK), Heidelberg, Germany; e German Cancer Research Center (DKFZ), Heidelberg, Germany; f Wecker Eye Center, Heilbronn, Germany

## Abstract

Preclinical Magnetic Resonance Imaging at high field strength offers the great advantage of combining anatomical information and very high resolution of down to 25 µm in mice and even higher resolutions in ex vivo settings. The presented data is Time of Flight MR imaging data using a tube phantom and a given flow-rate to determine the lower limit of the flow rate that is detectable with an experimental set-up and a specifically optimized 2D TOF sequence.

In this work we present data on a phantom study which shows the ability of Time of Flight MR Imaging to detect very low flow rates down to 25 µl/h at a velocity of 0.1 mm/s non-invasively in a phantom study.

## Specifications Table

**Table:** 

Subject area	*MR Imaging, Physiology, Biology*
More specific subject area	*Oncology, Plant biology, Engineering*
Type of data	*Table, MR Imaging data*
How data was acquired	*TOF MR Imaging, Bruker BioSpec 94/21 (Bruker, Ettlingen, Germany)*
Data format	*analyzed*
Experimental factors	*n.a*
Experimental features	We performed an experiment using a tube phantom and a given flow-rate in order to determine whether very low flow rates were detectable with 2D Time of flight MR Imaging sequence
Data source location	*n.a.*
Data accessibility	*The Data is within this article*

## Value of the data

• TOF MR imaging is useful to detect flow data at very low flow rates of water, proton based fluids or potentially other MR active nuclei as shown in a phantom using an optimized TOF-MRI procedure.
• Non-invasive detection of flow data is possible in biological or in other experimental settings with a technical background for characterizing flow patterns
• Compared to other flow sensitive techniques, such as optical particle tracing, this data can be acquired relatively fast and non-invasively which opens up the possibility to measure physiological or technical flow on very low rates in a variety of biological or technical applications.

## 1 Data

Preclinical Magnetic Resonance Imaging (MRI) at high field strength offers the great advantage of combining anatomical information and very high resolution of down to 25 µm in mice and even higher resolutions in ex vivo settings. Furthermore the possibility to combine the morphological with functional information, such as perfusion or diffusion in the same experiment [1] offers valuable information. The Time of Flight MRI (TOF-MRI) technique is very potent for the high resolution imaging of perfusion at very low rates in biological or technical structures. TOF-MRI is a non-invasive technique without the need to use contrast agents [2], [3], [4]. This makes it particularly useful to characterize vessel structures and their alterations in vivo for example in tumors before and during therapy [5], [6], [7].

**Fig. 1:**
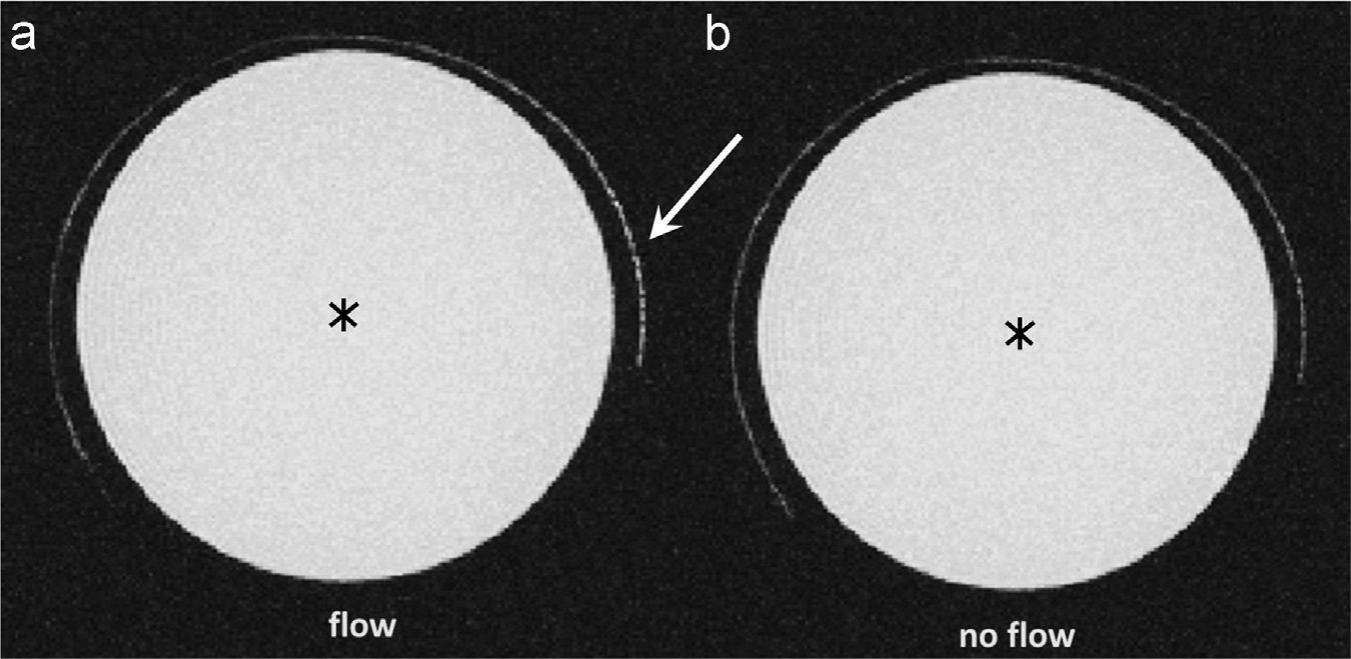
Image showing data of a Maximum Intensity Projection (MIP) of a TOF MR Imaging technique to visualize inflow. Imaging was performed under flow **(a)** and no flow **(b)** conditions. The arrow indicates the region where the flowing water (unsaturated spins) entered the imaging slice under flow conditions. The asterix indicates the small container filled with 20 ml of 2% Agarose was used to simulate the surrounding tissue and provide a better filling factor.

To test whether we can detect flow rates down to 25 µl/h we used a 0.28 mm diameter tube phantom connected to a perfusor pump (Harvard apparatus, USA) set it to its lowest possible flow rate and compared it to the same measurement with no flow at all (Fig. 1).

**Fig. 2:**
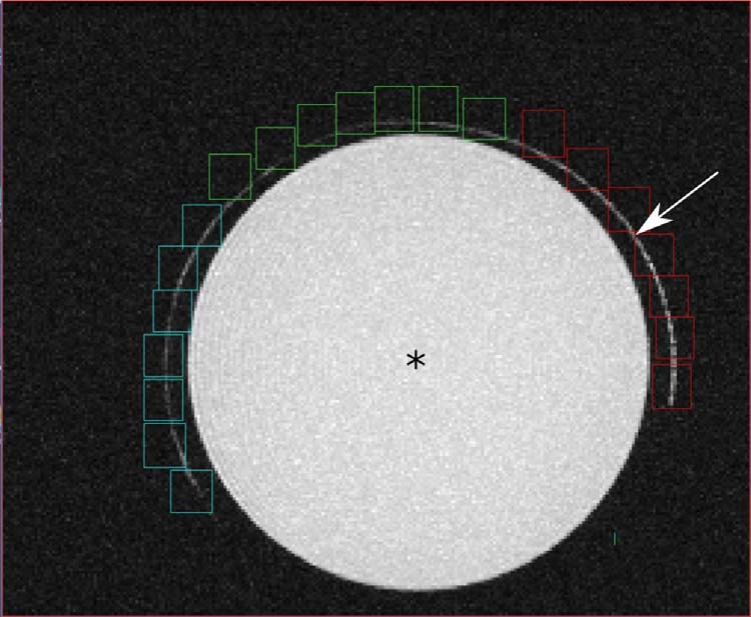
Image showing data of the quantitative evaluation of inflow using an MIP of a TOF MR Imaging technique. The arrow indicates the region where the flowing water (unsaturated spins) entered the imaging slice. The asterix indicates the small container filled with 20 ml of 2% Agarose was used to simulate the surrounding tissue, and vitreous body.

**Fig. 3:**
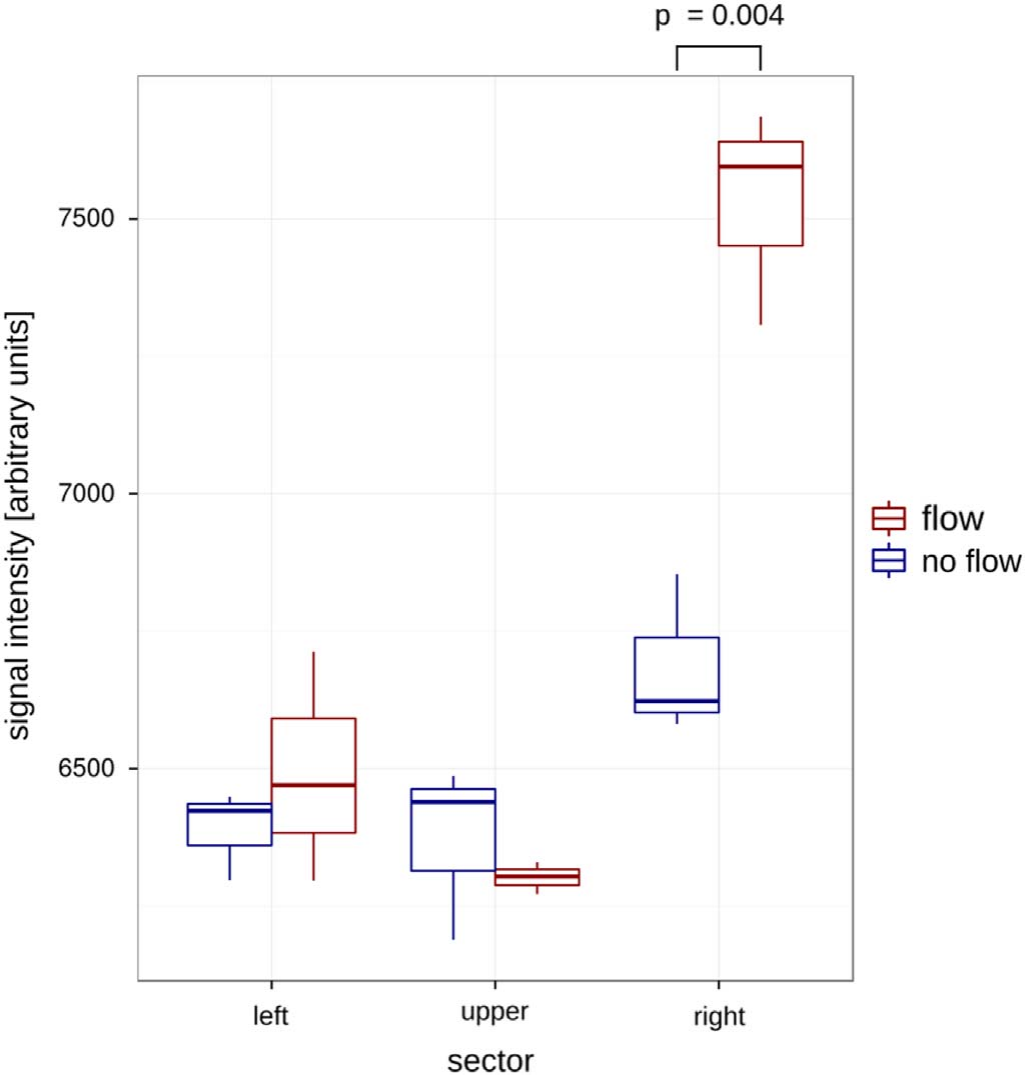
A significant (*p* < 0.01, two sample t-test) increase of signal intensities was detected in the region where the flowing water entered the imaging slice under flow conditions (red). Under no flow conditions (blue) no increase of the signal intensities was detected. Each experiment (flow and no flow conditions) was repeated three times. Right sector: red frames, upper sector: green frames, left sector: turquoise frames in Fig. 2.

Applying our optimized measurement protocol in this phantom the quantitative evaluation (Fig. 2) shows a significantly higher signal (Fig. 3) than the background noise from the no-flow condition control experiment.

## 2 Experimental design, materials, and methods

### 2.1 MR methods

TOF MR Imaging was performed using a 9.4 T preclinical magnetic resonance tomograph (BioSpec 94/21 USR, Bruker, Ettlingen, Germany) with Paravision 5.1 (Bruker, Ettlingen, Germany) using the following imaging Protocol: 1) high resolution T2 RARE sequence in coronal orientation that could be used as an overview imaging reference for 2) Time of Flight (30 thinly sliced 2D TOF MRI images to detect the flow patterns in the phantom setup.

The T2-RARE measurement in coronal orientation was used as reference for the consecutive TOF MRI images. Parameters for the high resolution T2-RARE image were as follows: FOV 4 cm × 4 cm, matrix 384 × 384 pixel, TR/TE 3540/36 ms, slice thickness 0.25 mm. The high resolution T2-RARE TOF MRI images were acquired in an axial plane with the same FOV and slice thickness as the high resolution T2-images to provide an identical geometry and in-plane resolution. The pixel matrix was 256 × 256. TR was set to a relatively high level (45.0 ms) allowing the unsaturated protons to travel into the imaging plane around the phantom and still generate a detectable MR signal despite a relatively slow flow. The flip angel (FA) was set to 90° to ensure maximum background suppression by enhancing the functional contrast of the active channels. Due to incomplete saturation there is residual signal left specifically with long measurement times.

### 2.2 Flow measurements

For the experimental setup, a phantom model was build: A small container filled with 20 ml of 2% Agarose was used to create a sufficient filling factor for the used MR coil. We wrapped around this a single half loop of PE tubing (inner diameter 0.28 mm) that spirals around the container. A 2D thinly sliced TOF-Sequence was used to detect the flow signal. In order to generate a constant and reliable flow, we used a preclinical injector (Harvard apparatus, USA) which we set to its lower limit of 25 µl/h that resulted in a calculated flow velocity of 0.1 mm/s within the tube. Altogether 3 measurements were performed under flow and no flow conditions. Following a Maximum Intensity Projection (MIP) postprocessing of the acquired image Stack, the TOF signal intensity was quantified within the tube lumen. Under no flow conditions an unspecific, residual background signal was detected. However, under flow conditions we obtained a weak, but significantly higher MR signal in the region where the flowing water (unsaturated spins) entered the imaging slice (*p* < 0.01, two sample *t*-test, Fig. 3). Due to the higher imaging signal in the flow condition we were able to detect a minimal flow velocity of 0.1 mm/s with a volume flow rate of 25 µl/h using our experimental setup.

### 2.3 Postprocessing

The Total imaging time for the experiment was 4 h and 19 min. TOF MR images were subsequently transferred from the acquisition software (Paravision 5.1) and exported into a Digital Imaging and Communications in Medicine (DICOM) – compliant format. In the next step the image stack was flattened using a MIP Z-projection (Fig. 1).

### 2.4 Quantification and comparison of flow

After postprocessing (see above), each MIP image was divided into three sectors and the sectors were aligned as shown in Fig. 2. The sectors were consecutively numbered in counterclockwise direction, starting with the sector where flowing water (unsaturated spins) entered the imaging slice. The mean signal intensity of all pixels in each sector was measured. The total TOF MR signal (sum of all pixel intensities in the particular sector) from each sector was averaged and compared to each other. The experiment was repeated 3 times (*n* = 3).

### 2.5 Data analysis

Statistical assessment was done using GNU R (R Core Team, 2014) and additional packages (Wickham, 2007, 2009, 2011). For comparison of the flow signal intensity between flow and no flow conditions a two-sample t-test was used and results were presented as mean ± SEM. (Fig. 3).
